# Author Correction: Cold-induced *Arabidopsis* FRIGIDA nuclear condensates for *FLC* repression

**DOI:** 10.1038/s41586-023-06523-5

**Published:** 2023-08-14

**Authors:** Pan Zhu, Clare Lister, Caroline Dean

**Affiliations:** John Innes Centre, Norwich Research Park, Norwich, UK

**Keywords:** Nuclear speckles, Gene silencing, Long non-coding RNAs, Plant molecular biology, Abiotic

Correction to: *Nature* 10.1038/s41586-021-04062-5 Published 3 November 2021

In the original version of this article, data showing the quantification of colocalization between FRI-GFP condensates and *FLC* signals were missing in the right-hand plots of Fig. 1g and 1h, with only the raw data provided in the source data. In both cases there is no co-localization, as discussed in the manuscript, so this correction does not influence any results or conclusions in the article. The figure and source data have now been updated in the HTML and PDF versions of the article.

