# Supplementary material for: Cold-induced Arabidopsis FRIGIDA nuclear condensates for FLC repression
Source: Nature. 2021 Nov 3;599(7886):657–61. doi: 10.1038/s41586-021-04062-5 (PMC8612926; doi:10.1038/s41586-021-04062-5)
Supplement: Supplementary file 1 — The original source images for the western blots. [file 41586_2021_4062_MOESM1_ESM.pdf]

nature portfolio

https://doi.org/10.1038/s41586-021-04062-5

# Supplementary information

# Cold-induced *Arabidopsis* FRIGIDA nuclear condensates for *FLC* repression

In the format provided by the authors and unedited

Supplementary Fig. 1 | The original source images for western blots

**Extended Data Fig. 5a**

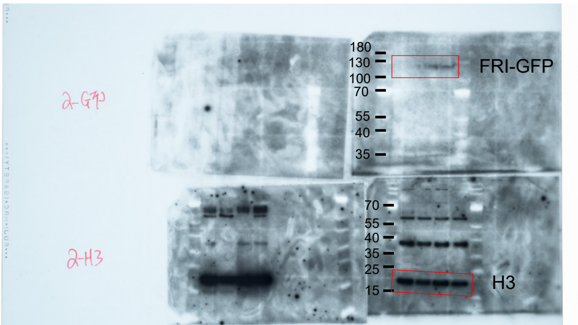


H3 was run on a a seperate gel

**Extended Data Fig. 5d**

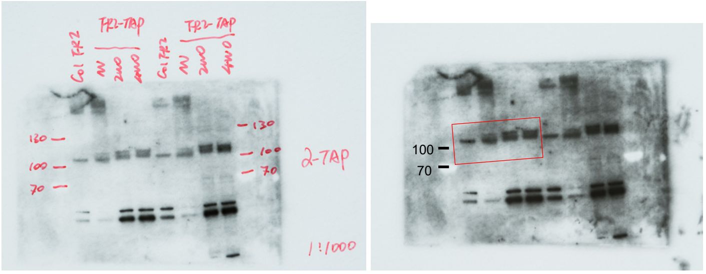


short exposure

long exposure

**Extended Data Fig. 6b**

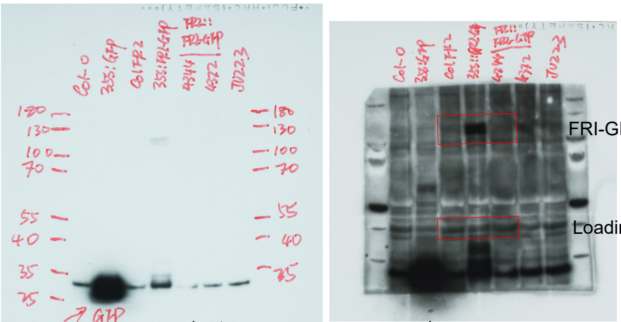


short exposure

long exposure

**Extended Data Fig. 6c**

FRI-GFP

Ponceau staining

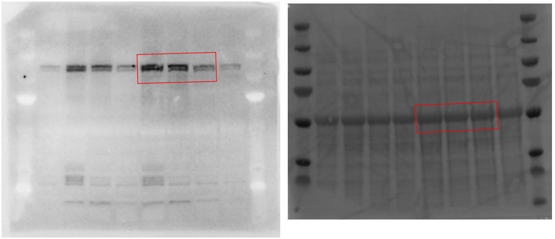

Membrane was stained for loading control after exposure

**Extended Data Fig. 6f**

0% Glucose

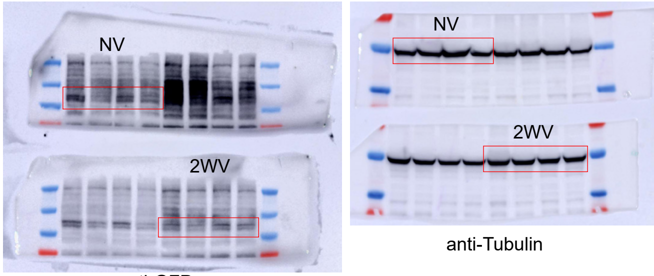


anti-GFP

anti-Tubulin

1% Glucose

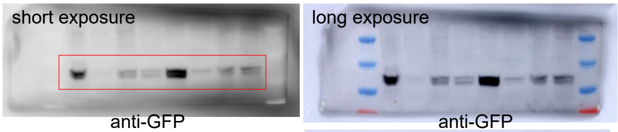


anti-GFP

anti-GFP

Tubulin was run on a same gel and membrane was cut for immnoblotting with different antibodies.

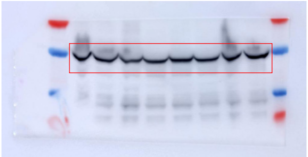

anti-Tubulin

**Extended Data Fig. 7d**

FRI-GFP

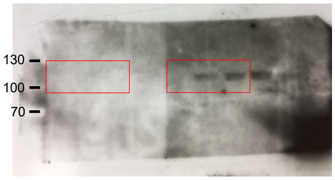


Ponceau staining

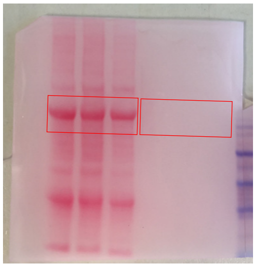

Loading was run on a a seperate gel
