# Supplementary material for: Cold-induced Arabidopsis FRIGIDA nuclear condensates for FLC repression
Source: Nature. 2021 Nov 3;599(7886):657–61. doi: 10.1038/s41586-021-04062-5 (PMC8612926; doi:10.1038/s41586-021-04062-5)
Supplement: Supplementary file 2 — Reporting Summary [file 41586_2021_4062_MOESM2_ESM.pdf]

nature portfolio

Corresponding author(s): Caroline Dean

Last updated by author(s): Aug 31, 2021

# Reporting Summary

Nature Portfolio wishes to improve the reproducibility of the work that we publish. This form provides structure for consistency and transparency in reporting. For further information on Nature Portfolio policies, see our Editorial Policies and the Editorial Policy Checklist.

## Statistics

For all statistical analyses, confirm that the following items are present in the figure legend, table legend, main text, or Methods section.

| n/a | Confirmed | |
|---|---|---|
| ☐ | ☒ | The exact sample size (*n*) for each experimental group/condition, given as a discrete number and unit of measurement |
| ☐ | ☒ | A statement on whether measurements were taken from distinct samples or whether the same sample was measured repeatedly |
| ☐ | ☒ | The statistical test(s) used AND whether they are one- or two-sided<br>*Only common tests should be described solely by name; describe more complex techniques in the Methods section.* |
| ☒ | ☐ | A description of all covariates tested |
| ☐ | ☒ | A description of any assumptions or corrections, such as tests of normality and adjustment for multiple comparisons |
| ☐ | ☒ | A full description of the statistical parameters including central tendency (e.g. means) or other basic estimates (e.g. regression coefficient) AND variation (e.g. standard deviation) or associated estimates of uncertainty (e.g. confidence intervals) |
| ☐ | ☒ | For null hypothesis testing, the test statistic (e.g. *F*, *t*, *r*) with confidence intervals, effect sizes, degrees of freedom and *P* value noted<br>*Give P values as exact values whenever suitable.* |
| ☒ | ☐ | For Bayesian analysis, information on the choice of priors and Markov chain Monte Carlo settings |
| ☒ | ☐ | For hierarchical and complex designs, identification of the appropriate level for tests and full reporting of outcomes |
| ☒ | ☐ | Estimates of effect sizes (e.g. Cohen's *d*, Pearson's *r*), indicating how they were calculated |

*Our web collection on statistics for biologists contains articles on many of the points above.*

## Software and code

Policy information about availability of computer code

| | |
|---|---|
| Data collection | For LCMS analysis, an Orbitrap Fusion™ Tribrid™ mass spectrometer (Thermo Fisher Scientific) equipped with an UltiMateTM 3000 RSLCnano LC system (Thermo Fisher Scientific) was used; all QPCR reactions were run with LightCycler® 480; all confocal images and movies were collected on Zeiss LSM780 confocal microscope, Zeiss LSM880 confocal microscope and Zeiss Elyra PS as indicated in the Methods; Western blot signal was captured in ImageQuant LAS 500. |
| Data analysis | Mascot server 2.7 (Matrix Science), Scaffold 4 (4.8.4), Microsoft Excel (version 2102, 64-bit), GraphPad Prism 7, Fiji-ImageJ 1.52i (Java 1.8.0_172, 64-bit) ), and Zen Black and Zen Blue (2012). Web service: PlotsOfData (https://huygens.science.uva.nl/PlotsOfData/) and IUPred2A (https://iupred2a.elte.hu/). |

For manuscripts utilizing custom algorithms or software that are central to the research but not yet described in published literature, software must be made available to editors and reviewers. We strongly encourage code deposition in a community repository (e.g. GitHub). See the Nature Portfolio guidelines for submitting code & software for further information.

## Data

Policy information about availability of data

All manuscripts must include a data availability statement. This statement should provide the following information, where applicable:

- Accession codes, unique identifiers, or web links for publicly available datasets
- A description of any restrictions on data availability
- For clinical datasets or third party data, please ensure that the statement adheres to our policy

Full lists of mass spectrometry are provided as Supplementary Table 1. Uncropped images of western blots are provided as Supplementary Figure 1. Other raw

images that support the findings of this study are available at Science Data Bank http://www.doi.org/10.11922/sciencedb.01119. Source data for quantitative analysis are provided with this paper.

# Field-specific reporting

Please select the one below that is the best fit for your research. If you are not sure, read the appropriate sections before making your selection.

☒ Life sciences ☐ Behavioural & social sciences ☐ Ecological, evolutionary & environmental sciences

For a reference copy of the document with all sections, see nature.com/documents/nr-reporting-summary-flat.pdf

# Life sciences study design

All studies must disclose on these points even when the disclosure is negative.

| | |
|---|---|
| Sample size | The sample size and the results of statistical analyses are described in the relevant figure legends. No statistical approach was used to predetermine sample size. Sample sizes were determined based on previous publications on similar experiments. The determined sample size was adequate as the differences between experimental groups was significant and reproducible.<br>Previous publications considered to determine sample size:<br>IP-MS analysis (https://doi.org/10.1038/s41586-019-1165-8)<br>RNA expression analysis (https://doi.org/10.1038/s41586-020-2485-4)<br>Microscopy and image quantification (https://doi.org/10.1038/s41586-019-1165-8; https://doi.org/10.1038/s41586-020-2485-4)<br>FRAP (Doi: 10.1007/978-1-4939-7318-7_26; https://doi.org/10.1038/s41586-019-1165-8)<br>Time-lapse imaging (https://doi.org/10.1038/s41586-019-1165-8)<br>Drug treatment (https://doi.org/10.1038/s41586-020-2485-4)<br>smFISH and immunofluorescence (DOI:10.21769/BioProtoc.2240; https://doi.org/10.1038/ncomms13031)<br>CHIP and qPCR (DOI: 10.1126/science.aan1121)<br>Western blot (https://doi.org/10.1038/s41586-019-1165-8; https://doi.org/10.1038/s41586-020-2485-4)<br>RNA-IP (https://doi.org/10.1038/s41586-019-1165-8) |
| Data exclusions | No data was excluded from analysis. |
| Replication | All key experimental findings were reproduced in more than three independent biological repeats with multiple technical replicates. All data except for the immunoblots are representative of at least three independent biological replicates. The immunoblot data are representative of two independent biological replicates. Similar results were obtained from independent biological replicates. Main conclusions were confirmed in different assays, including genetic assays in different mutant backgrounds or overexpression, bioimaging and immunoblots with transgenic lines carrying different tags. |
| Randomization | Plants of different genotypes were grown side by side to minimize unexpected environmental variations during growth and experimentation. Different treatments were carried out in parallel, with minimum covarying factors. Seedlings at the same developmental stage were collected and assessed randomly for each genotype/treatment.<br><br>For IP-MS, RNA-IP and CHIP, multiple seedlings were randomly collected from different plates for each replicate. For RNA expression/protein accumulation analysis and bio-imaging, multiple, randomly selected plants were collected from a plate for each replicate. |
| Blinding | Blinding was not applicable for this study because plants grown at different temperature conditions need to be collected at different growing time points and require specific handling temperatures. |

# Reporting for specific materials, systems and methods

We require information from authors about some types of materials, experimental systems and methods used in many studies. Here, indicate whether each material, system or method listed is relevant to your study. If you are not sure if a list item applies to your research, read the appropriate section before selecting a response.

## Materials & experimental systems

| n/a | Involved in the study | |
|---|---|---|
| ☐ | ☒ | Antibodies |
| ☒ | ☐ | Eukaryotic cell lines |
| ☒ | ☐ | Palaeontology and archaeology |
| ☒ | ☐ | Animals and other organisms |
| ☒ | ☐ | Human research participants |
| ☒ | ☐ | Clinical data |
| ☒ | ☐ | Dual use research of concern |

## Methods

| n/a | Involved in the study | |
|---|---|---|
| ☒ | ☐ | ChIP-seq |
| ☒ | ☐ | Flow cytometry |
| ☒ | ☐ | MRI-based neuroimaging |

## Antibodies

Antibodies used

For Western blot:
Primary antibodies: anti-GFP (Roche, 11814460001, a mixture of clones 7.1 and 13.1, dilution 1:2,000), anti-TAP (Thermo Fisher, CAB1001, polyclonal, dilution 1:1,000), anti-H3 (Abcam, ab1791, polyclonal, dilution 1:5,000), anti-Tubulin (Merck Sigma-Aldrich, T5168, monoclonal, dilution 1:4,000); secondary antibodies: Mouse IgG HRP Linked Whole Antibody (GE, NXA931; 1:20,000), Rabbit IgG HRP Linked Whole Antibody (GE, NA934; 1:20,000).
For immunofluorescence:
Primary antibodies: anti-c-Myc (Sigma, M5546, clone 9E10, dilution: 1:125), anti-GFP (Abcam, ab290, dilution: 1:500), anti-U2B'' (4G3, a gift from Prof. Peter Shaw, originally obtained from Euro-diagnostica B.V., Apeldoorn, Netherlands, dilution: 1:20); secondary antibodies: Alexa Fluor 488 anti-Rabbit secondary antibody (Thermo Fisher, A-11008, dilution: 1:200) and Alexa Fluor 555 anti-mouse secondary antibody (Thermo Fisher, A-21424, dilution: 1:200).
For immunoprecipitation:
anti-GFP (Abcam, ab290, dilution: 1:400), GFP-Trap magnetic agarose beads (Chromotek, GTMA-20, dilution: 1:40).

Validation

anti-U2B'', 4G3, a gift from Prof. Peter Shaw, was validated by many previous studies for marking Cajal bodies.

Validation statements and relevant citation of all the other antibodies are available from the manufacturers and most of them are also validated in this manuscript:

anti-GFP (Roche, 11814460001)-https://www.sigmaaldrich.com/catalog/product/roche/11814460001?lang=en&region=GB; also validated in Extended data Figs. 5a, 6b, and 7d where non-tagged FRI shows no bands.
anti-TAP (Thermo Fisher, CAB1001)-https://www.thermofisher.com/antibody/product/TAP-Tag-Antibody-Polyclonal/CAB1001; also validated in Extended data Fig.5d with non-tagged FRI showing no FRI-TAP band.
anti-H3 (Abcam, ab1791)-https://www.abcam.com/histone-h3-antibody-nuclear-marker-and-chip-grade-ab1791.html; anti-Mouse IgG (GE, NXA931)-https://www.sigmaaldrich.com/catalog/product/sigma/genxa9311ml?lang=en&region=GB&gclid=CjwKCAiA9bmABhBbEiwASb35V0Lm5FrPvLOvJgcZsOjzs756hT2PxI9x1HuHc_LyTQPZO_UXApnPWRoC5rMQAvD_BwE;
anti-Tubulin (Merk Sigma-Aldrich, T5168)-https://www.sigmaaldrich.com/GB/en/product/sigma/t5168?context=product; anti-Rabbit IgG (GE, NA934)-https://www.sigmaaldrich.com/catalog/product/sigma/gena9341ml?lang=en&region=GB&gclid=CjwKCAiA9bmABhBbEiwASb35Vw9OIG58qJ0JS9_VrsKOXrp6JXUTUKV8iLDXc61BRdXi_CByayrNBRoCHjsQAvD_BwE;
anti-c-Myc (Sigma, M5546)-https://www.sigmaaldrich.com/catalog/product/sigma/m5546?lang=en&region=GB; also validated in Extended data Fig. 2e with non-tagged FRI showing no signal.
anti-GFP (Abcam, ab290)-https://www.abcam.com/gfp-antibody-ab290.html; also validated in Extended data Fig.3h with negative control showing no signal and in Extended data Fig. 7d-f with no enrichment in negative control.
Alexa Fluor 488 anti-Rabbit secondary antibody (Thermo Fisher, A-11008)-https://www.thermofisher.com/antibody/product/Goat-anti-Rabbit-IgG-H-L-Cross-Adsorbed-Secondary-Antibody-Polyclonal/A-11008;
Alexa Fluor 555 anti-mouse secondary antibody (Thermo Fisher, A-21424)-https://www.thermofisher.com/antibody/product/Goat-anti-Mouse-IgG-H-L-Highly-Cross-Adsorbed-Secondary-Antibody-Polyclonal/A-21424;
GFP-Trap magnetic agarose beads (Chromotek, GTMA-20)-https://www.chromotek.com/products/detail/product-detail/gfp-trap-magnetic-agarose/; also validated in Fig. 3b and Extended data Fig. 9h with no enrichment detected in negative control.
